# How simulated patients contribute to student learning in an authentic way, an interview study

**DOI:** 10.1186/s41077-023-00277-w

**Published:** 2024-01-11

**Authors:** Annelies Lovink, Marleen Groenier, Anneke van der Niet, Heleen Miedema, Jan-Joost Rethans

**Affiliations:** 1 Department of Technical Medicine, University of Twente, Utwente, Hallenweg 5, Enchede, 75522 NH the Netherlands; 2 Department IQ Healthcare, Radboud University Medical Center Nijmegen, Nijmegen, the Netherlands; 3 Skillslab, Faculty of Health, Medicine and Life Sciences, Maastricht University, Maastricht, the Netherlands

**Keywords:** Simulated patients (SPs), Medical communication, Student learning, Meaningful learning, Positioning theory

## Abstract

**Introduction:**

Simulated patients (SPs) play an instrumental role in teaching communication skills and enhancing learning outcomes. Prior research mostly focused on the SP’s contribution to students’ learning outcomes by providing feedback afterwards. A detailed understanding of the contribution of the SP during SP-student encounters is currently lacking although the majority of the interaction between SPs and students occurs during the SP-student encounter. Therefore, this study focuses on how SPs see their contribution to meaningful student learning experiences during SP-student encounters.

**Methods:**

We interviewed fifteen simulated patients from one institution. We explored their perspectives on meaningful learning experiences during SP-student encounters through in-depth, semi-structured interviews and analyzed using thematic analysis.

**Results:**

SPs view their contribution to meaningful student learning during SP-student encounters from two perspectives. A collective perspective as a member of the community of SPs and an individual perspective. From the collective perspective, SPs believe that the fact that students deal with multiple varied SP-student encounters over time is of value for meaningful learning. From the individual perspective, we noticed that SPs think, act, and react from three different positions. First, as the patient in the role description, second, as a teaching aid and third, as an individual with personal experiences, beliefs, and values. SPs mentioned that the ratio between these different positions can vary within and between encounters.

**Conclusions:**

According to SPs, we should value the variation between SPs, thereby creating meaningful variation in authentic interactions in SP-student encounters. SPs should be allowed to act and react from different positions during SP-student encounters, including their role description, as teaching aid, and based on their own experiences. In this way, SP-student encounters are optimized to contribute to meaningful student learning through authenticity.

## Background

To improve health outcomes for patients and to enhance patient and professional satisfaction, good communication between patients and professionals is essential in high-quality healthcare [1]. Communication skills are among the fundamental competencies taught in medical educational programs.

### Benefits of simulated patients

Simulated patients (SP) have been involved extensively in teaching and assessing communication skills of healthcare students since their introduction by Barrows in 1964 [2]. SPs are defined as laypeople or actors who have been trained to portray a patient with a specific condition in a realistic way [3, 4]. Existing research on SPs has focused primarily on how to use SPs as effectively as possible, the impact of SP feedback on students’ communication skills, the cognitive demand on SPs and SP perspectives on becoming and being a SP [5–7].

SPs facilitate students to apply and integrate theoretical knowledge into practice, thereby challenging them to think critically, solve problems and increase their clinical judgment and communication skills [7, 8]. Compared with real patients, SPs enable trainees to experiment and try out different approaches to communication skills and offer an opportunity to standardize and customize communication skills training to specific learning objectives [9]. Compared with peers, SPs enable students to be more fully immersed in the reality of clinical situations [8]. Effective SPs are competent in portraying a patient according to a role script, offering a certain level of standardization. SPs may vary in levels of required standardization in portrayal, which can depend on factors such as educational objectives [10]. Also, SPs are trained to observe students’ behavior and recalling the encounter accurately to give feedback to the student afterwards [11–13]. Studies show that students highly value feedback from a patient perspective provided by the SP [5, 11]. However, feedback from SPs is always given *after* the encounter, while in general the amount of time that is dedicated to interaction between SPs and students is longer *during* the consultation. Considerable research has already been conducted on SPs and their inherent value for student learning, providing insights into effective ways of involvement and giving feedback, see for example Lane and Rollnick [8], Leonardi [12] and Stillman et al. [14].

Nevertheless, the specific dynamics within the SP-student encounter that facilitate student learning still lack clarity. It is therefore necessary to better understand what exactly happens during the SP-student encounters and how students learn from these interactions in a meaningful way.

### Meaningful learning and feedback-in-action

Meaningful learning can be described as learning that is well anchored and integrated into the cognitive structure of learners, in contrast to rote learning such as reproduction-oriented learning [15]. The interaction between the student and the SP during an encounter can facilitate meaningful learning [15, 16]. SPs can provide implicit feedback-in-action, which can initiate a process of reflection-in-action [16].

This indicates that it is not only feedback afterwards that is valuable for meaningful student learning but also the reactions of the SP during the SP-student encounter. Meaningful learning thus might occur more than we are aware of during the SP-student encounters, however, it is unclear how SPs contribute to this while they are simultaneously portraying a patient.

### The contribution of the SP to meaningful learning

A previous study about meaningful learning during SP-student interaction from a student’ perspective showed that the authentic reaction of the SP during the encounter is important to facilitate meaningful learning [16]. This authentic reaction of the SP provides students with feedback during the SP-student encounter, so-called feedback-in-action [16]. It is important to notice that feedback-in-action differs from so-called in-role-feedback [11]. In-role-feedback is a way of giving feedback after the encounter where the SP remains in the role, whereas feedback-in-action is the reaction of the SP *during* the encounter, integrated into the role-play. Students described that feedback-in-action provided by the SP contributed not only to their communication skills but also to their identity development on a personal and professional level [16].

However, to our knowledge the SPs’ perspective on their contribution to student learning *during* the encounter has not yet been studied. Understanding the contribution of SPs during the encounter itself is crucial because it provides insights into the immediate impact the SP and SP-student interaction can have on student learning. While post-encounter feedback is valuable, gaining a more in-depth understanding of the SP contribution during the encounter can enhance the quality of the student learning. The SP actively participates in the educational process both during and after the SP-student encounter. However, due to their unique role during the interaction, the SP has a unique perspective on student learning. *Research question.*

Therefore, our research question is: What is the perspective of simulated patients on their contribution to meaningful student learning during SP-student interaction? By better understanding the perspective of the SP on their contribution to meaningful student learning during the SP-student encounter, SP-mediated learning could be enhanced.

## Methods

### Study design

#### Theoretical framework

An interpretivist research paradigm and a qualitative approach were adopted [17]. We conducted semi-structured individual interviews with 15 SPs to facilitate an in-depth exploration of the SP perspective. We explored the SP perspective on their contribution to meaningful learning experiences during SP-student encounters and used thematic analysis to analyze the data [18].

#### Setting

This study was conducted at the undergraduate Technical Medicine program of the University of Twente (the Netherlands) and ethically approved by the Netherlands Association of Medical Education (NMVO, NERB number 1050). The 40 SPs at the University of Twente vary in SP experience from 1 to 15 years, work on a non-contact basis and can indicate their availability per half year. They range in age from 30 to 70 years with a male/female ratio of 30/70. They are all lay people who have been trained to portray a patient realistically and to give feedback from the patient’s perspective. The majority of SPs are involved in SP-student encounters more than 15 times a year.

The student communication program involves 15 SP-student encounters per student throughout the three-year bachelor curriculum, in which students practice basic skills, such as asking open questions, to advanced communication skills, such as breaking bad news.

#### Participants

We used purposive sampling to identify participants. Eligible participants included SPs who had a minimum of three years of experience as an SP. This criterion ensured that they had multiple experiences with SP-student encounters and had the expertise needed to reflect on their contributions. They were randomly approached by email to participate. We interviewed a total of *n* = 15 SPs between February and June 2020, nine women and six men, aged between 30 and 70 years.

#### Data collection

We developed a semi-structured interview guide (Appendix) to gain insight into the contribution of SPs to meaningful student learning. Based on a pilot interview, the interview guide was refined (see Appendix). We asked questions such as: *Sometimes the learning is more in the little moments during the consultation. What do you think are important moments in consultations? What can the student learn from these moments?*

One Technical Medicine master student conducted all 15 interviews in Dutch (lasting 40 to 60 min). We selected this master student to ensure that her role as interviewer did not interfere with her role as a student involved in the bachelor communication training. All interviews were audio recorded and transcribed verbatim. The other researchers only had access to the anonymized data.

### Analysis

**Fig. 1:**
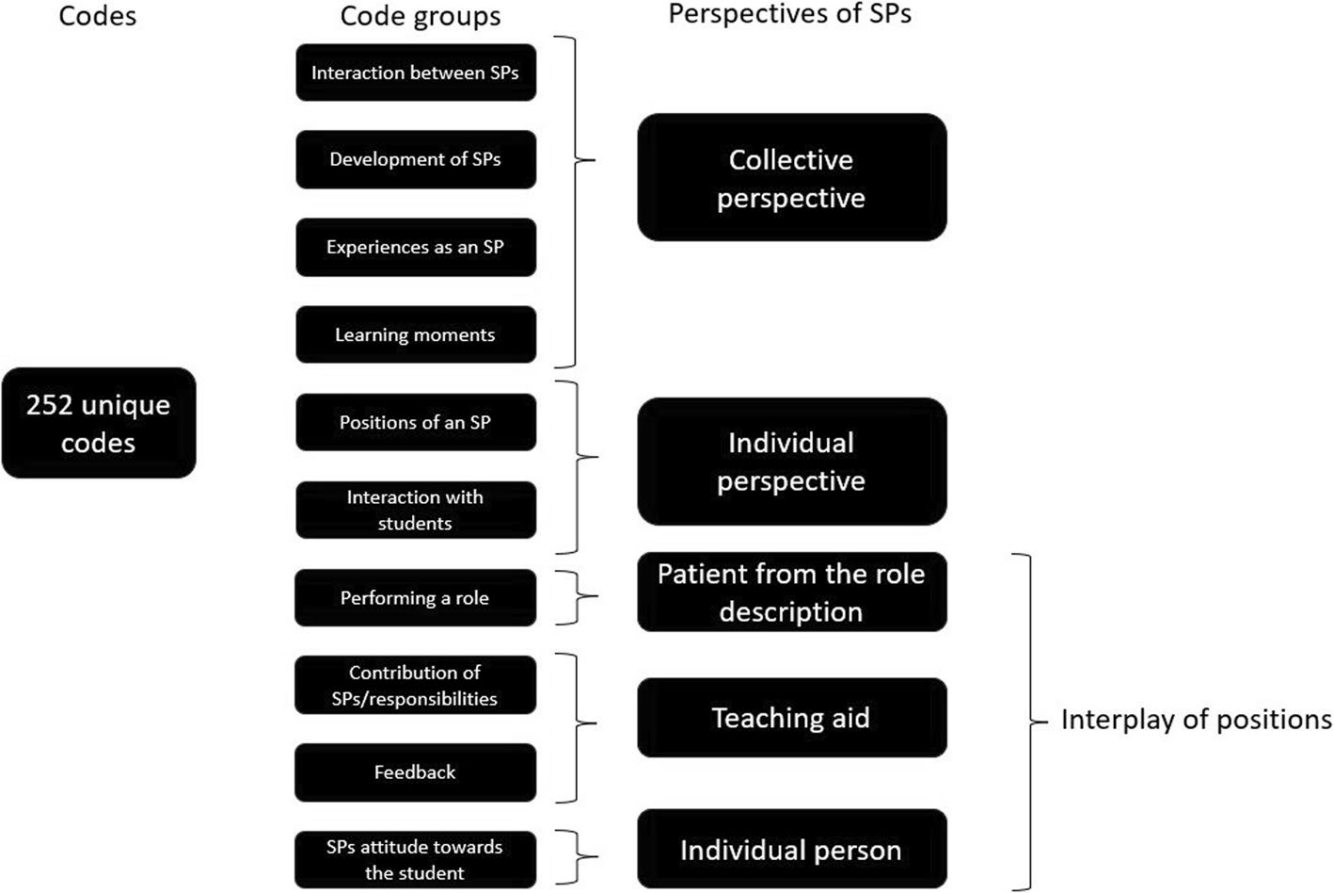
Overview of the code groups identified by the researchers in relation to the different perspectives of SP

Thematic analysis was used to analyze the data, and search for themes and patterns (18). Three researchers (AL, MG, AvdN) independently coded the first three interviews applying general principles of open coding, using the Atlas.ti. software program. Codes were identified in individual interviews by repeated reading and constant comparison of the individual interviews with the research question. Data were reviewed jointly followed by collaborative discussion among all researchers about the appropriateness of the codes. Meetings to compare and refine the analysis occurred after coding every three to four interviews. Codes and categories were discussed and clarified before the interviews were re-read and re-analyzed using the final coding guide. Saturation was reached after 14 of 15 interviews [19]. Figure 1 provides an overview of the code groups identified by the researchers and how they are related to the results.

### Reflexivity and research team

All authors adopted a reflexive attitude to discuss their initial observations of the data in relation to their own interests and biases. All researchers discussed reflexivity to identify their potential biases and presuppositions. They considered their own occupational roles and how these might affect their initial reading of the data. As a result of these discussions, the main researcher deliberately refrained from conducting the interviews due to her close involvement with the SPs. To minimize potential influence on SPs’ responses, interviews were conducted by a Technical Medicine master student.

In the spirit of reflexivity, we provide the authors’ relevant backgrounds. Researcher and interviewer AL is a lecturer and SP educator at the Department of Technical Medicine with 14 years of experience working with SPs. MG is a lecturer and researcher at the Department of Technical Medicine with substantial experience in human and non-human simulation education. During the time of this study HM was the director of education for Technical Medicine at UT. She designed the Technical Medicine educational program. AvdN has extensive research experience using qualitative methodologies in the field of medical education. JR is a professor in the field of human simulation and has worked with SPs since 1985.

## Results

We identified two perspectives from which SPs reflect on their contribution to meaningful student learning during the encounter: a collective perspective as a member of the community of SPs and an individual perspective. In the following sections, we describe these perspectives separately and provide context by presenting quotes from a variety of SPs as indicated by SP#.

### The collective perspective

#### Collective impact

When asked about their personal contribution to meaningful student learning, frequently SPs did not link their contribution directly to themselves but to the collective of simulation patients which they are part of the following:

No, not personally. I just think the simulation patient in general does. But whether they (the students) learn more from me than from another…. I do not believe so, no, no. (SP1)

And the rest depends on, well… it is integrated in being a simulated patient and not being me personally. (SP7)

#### Value of variation among SPs

SPs indicated that the variation of SPs and the fact that students have multiple and varied SP-student encounters [besides their own individual role-playing] are of great value for meaningful student learning.

By having a different simulated patient each time, a different situation each time, each time they [students] take part they can think: ‘o, that lady reacted this way but that does not necessarily mean.... because the other patient reacted in another way’. […] So, I think it is mostly due to the differences. If I were the only simulated patient here, they would not learn so much. But because of the differences each time between people. I think they can learn a lot from that. (SP1)

SPs repeatedly described that the personalities of the different SPs are reflected in various role portrayals. They mentioned this diversity as an added value for meaningful student learning:

That’s also the beauty of it, the diversity of the simulated patients. Although you try to play it all in a similar way, we are all different. (SP5)

To summarize, SPs considered student learning as an ongoing process to which SPs contribute over time, as part of their membership in the community of SPs. They indicated that they contribute to meaningful student learning from a collective perspective.

### The individual perspective

Although SPs feel they are part of a collective, they also bring their own individual perspective. We identified three interacting positions in this individual perspective: as a patient from the role description, as a teaching aid, and as an individual person. Firstly, we describe the three different positions separately; thereafter, we describe how these interact with each other during SP-student encounters.

#### SP as a patient from the role description

SPs are supposed to play the role that is assigned to them beforehand. They can prepare for this role by reading and practicing the role description. SPs described that they stick to the role and assignment as much as possible. They described this as being the patient:

I’m really… I try to be a patient as much as possible anyway. Hey, I have pain in my leg, I have a sore arm, I’m afraid of surgery. I try to be a patient as much as possible. [SP3]

I do try to stick to the role. If it says keep my distance, I keep my distance. Be the patient. I am always the patient. (SP7)

SPs are aware of the learning objective of a role description in the communication program:

But that’s why it’s so nice that you do indeed get a fully developed case. In which it is said very clearly: this is what is wrong with you, this is your character, this is what your expectations are, and this is what happens, or what has got to happen in that... in that… Yes I focus on that. I stick to that as much as possible. And that is, to my point of view at least, predetermined by the teacher to practice certain aspects. (SP3)

#### SP as a teaching aid

SPs described their own position as supportive of the established curricular goals. They also described that their role differs from the teacher’s role.

The teachers are the ones who teach, and they decide what needs to be done, how they [students] are assessed. And I do not have to take over that role. I always try to keep that clear for myself. You are not the teacher, you are just used for this part, and you have to stick to your task. (SP8)

Even when SPs have substantive medical knowledge from their own other or former professional occupation, they are well aware of their position as SP:

You’re not a teacher, you’re really a simulated patient. So professionally, I think you have to be very careful. Because of my own profession, I have some [medical] knowledge, but that’s not my role here, so I do not draw on that. (SP15)

This SP managed to put it very conscisely:

Yes I have… um…. I’m just a means to an end. (SP14).

Taken together, we refer to the position in which SPs place themselves in a supportive role in relation to the identified learning goals in terms of SPs as a teaching aid.

#### SP as an individual person

In addition to the role description and teaching aid, the SP as a person naturally resonates. Experiences of the SP as a patient in real life and preferences of how an SP wants to be addressed as a person shape the SP’s reaction during student-SP encounters:

And I have experienced very bad consultations too many times, or doctors who said very strange things. And that’s what I want to, that’s …eh…. What I try to avoid. So, that in all situations they [students] maintain appropriate communication…. (SP2).

Personal beliefs and motivations are also mentioned by the SPs to influence SP-student encounters:

And I do try to make it clear, that they [students] really try to empathize with the patient. And that is very important. And if I can contribute a little bit to that, then I’m happy. (SP2)

We argue that it is possible that these beliefs and motivations affect how SPs play their role, which may not always be in line with the opinion of the teacher:

And that did not really work out the way it was supposed to. Then one of the teachers said to me: Yes, but you were also a bit too honest and too sweet with that student. Then I think, yes, but I want to make something of it. If it is better at that moment to try to be a bit more amicable towards each other then I don’t think that is such a bad thing. (SP2)

On the other hand, SPs also described a reflective attitude on a personal level towards their contribution during the interaction with the student.

And I realize that my mission is to do as much as possible so that in real life students still learn how to interact with patients. How do I react to things? How am I doing? How is the patient? Yes, that is my goal to be able to contribute to that. (SP11)

Additionally, the actual SP’s personal family circumstances are also cited by SPs as affecting their attitude towards the student, for example, when a SP is familiar with the age group of the students:

My youngest is 27, so in that sense I do know their [students] perception. And I also know what being a student means. I also understand that if you have a party the night before, that you can be a bit wobbly. I also think that’s part of students’ lives. I think that’s why I can be a bit more forgiving than some others. (SP7)

### The interplay of positions

#### Mix of positions

The three positions described above interrelate to each other and, according to the responses of the SPs, coexist simultaneously during SP-student encounters. SPs described that there is a mix of positions during the encounter from which the SP thinks, acts, and reacts during SP-student encounters. As this SP explained:

I think that’s a mix. You have a role, but you are yourself [...] And of course yourself always comes through, that can’t be avoided. But because you get extensive information up front [role description] that highlights different aspects, you can empathize with the role very well. Within the context of who you are yourself, of course. (SP3)

#### Individual person and patient from the role description

Most SPs find it inevitable that the positions as a person and patient from the role description interfere with each other. SPs mentioned that is easier to play a role when the role description and their own personality are more in line with each other.

I do see myself as the patient from the role description. But there’s a bit of my own personality in that, of course. I also find that I often play a role that kind of fits me. I’m not an actress, I’m just someone who does this.... I’m not trained for this. We have training sessions from time to time but I try to do it as well as I can. I do notice that some roles suit you more than others and fit you better. And that I sometimes give a different interpretation to something than another simulation patient because I’m just a different person. (SP8)

#### Teaching aid and individual person

The same applies to the interplay between the position as teaching aid and individual person during encounters. SPs mentioned that they want to stick to the assignment but also direct the interaction based on their own beliefs.

It always depends on the assignment to what extent you have to stick to the role. And if it’s very strict, like well if the student isn’t able to comfort you…. Well if that’s the assignment, then I’ll stick to my role. But if there is some space, then I’m willing to try a side path, to help the students get back on their feet a little bit. But that very much depends on what I’m allowed, because I think the role is guiding. (SP7)

## Discussion

In this qualitative study, with an interpretivist research paradigm [17], the SPs’ perspectives on meaningful student learning concerning communication skills during SP-student encounters were explored. SPs considered student learning as an ongoing process to which SPs contribute over time. When asked about their contribution, SPs primarily stated that their contribution is a collective one: the variation between SPs and the diversity of consecutive SP-student encounters are of value for meaningful student learning. There appeared not only to be variation in thinking, acting, and reacting between SPs, SPs themselves also showed variation in the way they think and behave within their individual SP-student encounters. This is the result of the different positions that are at play. This study enhances our comprehension of meaningful learning during SP-student interaction, building upon a prior study that examined the students’ perspective [16].

### SPs’ positions

Our results showed that SPs may react from three different positions during specific SP-student encounters. The positions vary in relevance during every SP-student encounter. First, SPs can react as the patient from the role description. Second, SPs can react as teaching aids. Finally, SPs may also react based on their own experiences. Because of these different positions and the variation between SPs, the reactions of SPs during every SP-student encounter create unique learning experiences.

These results are partly in line with the study of Sullivan et al. and the study of Sargeant et al. [20, 21]. Sullivan et al. focused primarily on how different persona matter for feedback afterwards and described that the SP role involved managing more than one persona [20]. For example, while portraying the patient, SPs also took the role of assessor to remember details to discuss during feedback. In alignment with this perspective, our study reveals a nuanced mix of positions that SPs can take during an encounter, shaping their actions and responses. This enriches our understanding of how personal beliefs and motivations can resonate already during the encounter. Personal beliefs of SPs are also clearly present in the three positions described by Sargeant et al. [21] in a study on student professional behavior, applying positioning theory to examine how SPs position themselves in relation to students. This theory asserts that individuals will place themselves and others within institutional or individual frames of references [21]. Sargeant et al. found that SPs make sense of their positions by drawing on their own social representations as patients, trainers, but also as parental figures [21]. These three social representations together might best be compared to our position of the SP as an individual person, where personal experiences and beliefs resonate during the SP-student encounters. Our study builds upon the research of Sargeant et al. [21] and identified the various positions SPs may adopt during SP-student encounters and their impact on meaningful student learning.

This is a next step in unraveling the SP-student interaction, in which we try to understand why and how SPs change position during individual consultations and how that influences student learning. We suggest that the alternating influence of these positions of SPs, especially *during* the SP-student encounters, creates unique learning experiences that encourage reflection-in-action and thereby stimulate meaningful learning [16, 22].

### Freedom and authenticity

Based on our findings that SPs’ different positions during SP-student encounters are valuable for student learning, we recommend that SPs should have the freedom to act and react in an authentic way. There are no limitations on the various positions, allowing for flexibility in responding. This means, for example, that the SPs can react from the perspective of the patient, as well as incorporate their own personal emotion into the response, thereby making the reaction more authentic. Research shows that authenticity, defined as a reliable and accurate representation of reality, is one of the key aspects in creating a meaningful learning experience [16, 23].

Rystedt and Sjöblom were able to address authenticity from the learners’ point of view in a study on authenticity, realism, and learning in healthcare [24]. They described authenticity as an interactive achievement, something that participants create during the interaction. Starr et al., in a study on SP identity, described the interaction between the individual SPs’ real and simulated selves as bidirectional [25]. SPs’ real selves emerged while they were portraying a case, simultaneously being both the patient and themselves. Instead of seeing the influence of the real selves as a validity threat for standardization, they emphasized the positive influence of SPs real voices. Starr et al. argue that calling up true emotions and authentic verbal and nonverbal responses is what enables SPs to elicit humanistic and empathic responses from students [25]. SPs thereby provide a humanistic perspective in simulation [25].

While SPs themselves cannot be standardized due to their unique human qualities, SPs behavior can be calibrated along a continuum and within a bandwidth described as the Human Simulation Continuum Model [10]. This Human Simulation Continuum Model is a valuable tool in making decisions about the level of standardization [10]. Even in education settings that demand high levels of standardization, it is important to consider the effect of standardizing SPs’ behavior. Homogeneity in the reaction of SPs during teaching and assessment might not be the ultimate goal to aim for [10, 26]. To create a realistic and authentic learning experience based on an SP role description, it might be even better to pursue diversity, as this is a realistic reflection of reality. Nevertheless, standardization of context remains important to provide frameworks for SPs to act within.

For educators, it is a balancing act to strive for authentic situations while also providing an appropriate level of standardization. Establishing clear learning objectives supports defining the minimal level of standardization to ensure equal learning opportunities for students. With a lower level of standardization, there may be more freedom for the SP to react in an authentic way than when aiming for the highest level of standardization. This enables the creation of more meaningful learning experiences.

### Strengths and limitations

This study provides in-depth insight into SPs’ perspective on students’ meaningful learning during SP-student encounters based on a single data source from one group of SPs working in a technical medical school. We promoted the transferability of findings by describing the findings and the context in detail, explaining our sampling strategy, and discussing our findings in comparison to existing literature from different settings.

Credibility could be enhanced by exploring this topic from different perspectives, including the perspective of the SP educator or teacher. However, we used investigator triangulation and theory triangulation to promote trustworthiness.

Finally, we did not observe the SP student interactions in real-time or let SPs reflect on their contribution to a specific encounter directly afterwards. It would be beneficial to observe and analyze the behaviors and thoughts of the SP and student during and immediately after an SP-student encounter. This would lead to a more comprehensive understanding of the SP-student interaction and how this might be influenced by the different positions SPs can take on.

### Conclusion and future directions

According to SPs, we should value the variation between SPs and the variation in thinking, acting, and reacting of every SP during every SP-student encounter with regard to meaningful learning of communication skills. Different positions of SPs are at play even *during* individual SP-student encounters. We recommend that SPs should have the freedom to act and react from their different positions during SP-student encounters as in this way SP-student encounters are optimized to contribute to meaningful student learning. It can be helpful for SP educators to be familiar with the different positions, to create an optimal match between the SP, the role, and the educational goals.

Future studies should explore the balance between the unique characteristics of each SP to enable meaningful learning through authenticity and recognize the potential benefits of standardization. Meaningful variations in learning situations as described by Mylopoulos et al. [27] is a key educational approach that fosters deeper learning. Active learning paired with exposure to meaningful variation is essential for this deeper learning and multiple SP student encounters, each with unique characteristics, exemplify this approach in communication training [27, 28].

The perspective of the SP is important to consider when reflecting on students’ learning, as also described by Erici et al. [29]. Ongoing SP input, as described by Pritchard et al. [7] on SP programs, may benefit SPs and lead to higher quality of educational programs. To get a more detailed understanding of the actual SP-student interaction, it would be interesting to analyze the thoughts and behaviors of the SPs and students during SP-student encounters.

## Data Availability

The datasets used and analyzed during the current study are available from the corresponding author on reasonable request.

## Appendix

### Interview schedule for simulated patients about SP-student interaction

#### Semi-structured interview schedule

1. Introductory questions

1.1. How do you feel about being a simulation patient in our department?

1.2. How did you get started as an SP?

1.2.1. What was your motivation?

1.3. How do you experience yourself as an SP?

1.3.1. What kind of roles do you like to play?

1.3.2. How important is variety in different roles to you?

1.4. What do you enjoy most in being an SP?

1.5. What do you think are the important responsibilities of an SP?

1.5.1. How do you see this reflected in your work as an SP?

2. Questions about SP-student relationships

2.1. What is your contact with students like outside of role-playing? For example, prior to an exercise.

2.2. And how is your contact with students during role-playing?

2.3. How do you see the student during the consultation?

2.3.1. Do you see them as a student or as a beginning professional?

2.4. Throughout the consultation, there is always interaction. You respond to the student, and the student responds to you. What does that require of you?

2.4.1. Do you ever adapt your performance to the student or a situation? Or perhaps deliberately not?

2.4.2. If so, in what way?

2.4.3. Examples?

2.5. Is there an influence of age difference in the contact between you and the student?

2.5.1. If so, how do you deal with that?

2.6. Are there other differences that influence the contact between you and the student, for example, cultural differences, gender, power differences, or [life] experience?

2.6.1. If so, how do you deal with that?

2.7. Does the reason you once started as SP influence the contact with the student?

2.7.1. In what ways?

2.7.2. Has your motivation changed over time? In other words, do you view your commitment as an SP differently now than you did in the beginning? And how has that affected your contact with the student?

3. Questions about the student’s learning process

3.1. What do you think is important for students to learn from consultations with an SP?

3.1.1. How does the SP contribute to that learning?

3.1.2. How important is the SP’s contribution to that?

3.1.3. And how does that apply to you personally as SP?

3.2. Sometimes the learning is more in the little moments during the consultation. What do you think are important moments in consultations?

3.2.1. What can the student learn from from these moments?

3.3. What is the SP’s contribution to those moments, in your opinion?

3.3.1. And how does that apply to you personally as an SP, what do you contribute?

3.4. To what extent do SPs consciously create learning moments during an encounter or do they arise more naturally?

3.4.1. How does that apply to you?

3.5. To what extent do you already let the student know, during the encounter as a patient, whether the consultation is going well or not?

3.5.1. Does that happen consciously or unconsciously?

3.5.2. And how do you do that?

3.6. Can you give examples of moments when you thought ‘Yes, the student really learned something from that!’

3.6.1. Can you indicate what you contributed to those moments?

#### General questions

Participant number:

Age:

Gender:

Years of SP experience:

Date:

## Abbreviation

SP: Simulated patient
